# Regulating the Charge Migration in CuInSe_2_/N‐Doped Carbon Nanorod Arrays via Interfacial Engineering for Boosting Photoelectrochemical Water Splitting

**DOI:** 10.1002/advs.202300034

**Published:** 2023-04-23

**Authors:** Cheng Wang, Shengdong Sun, Hui Zhang, Jun Zhang, Chuanhao Li, Wei Chen, Shikuo Li

**Affiliations:** ^1^ Photoelectric Conversion Energy Materials and Devices Key Laboratory of Anhui Province Key Laboratory of Structure and Functional Regulation of Hybrid Materials (Anhui University) Ministry of Education School of Material Science and Engineering & School of Chemistry and Chemical Engineering Anhui University 230601 Hefei P. R. China; ^2^ School of Environmental Science and Engineering Sun Yat‐sen University Guangzhou 510006 P. R. China

**Keywords:** charge transfer kinetics, electron reservoirs, Interfacial interactions, N‐doped carbon layer, photoelectrochemical (PEC) water splitting

## Abstract

Regulating the charge migration and separation in photoactive materials is a great challenge for developing photoelectrochemical (PEC) applications. Herein, inspired by capacitors, well‐defined CuInSe_2_/N‐doped carbon (CISe/N‐C) nanorod arrays are synthesized by Cu/In‐metal organic frame‐derived method. Like the charge process of capacitor, the N‐doped carbon can capture the photogenerated electron of CISe, and the strong interfacial coupling between CISe and N‐doped carbon can modulate the charge migration and separation. The optimized the CISe/N‐C photoanode achieves a maximum photocurrent of 4.28 mA cm^−2^ at 1.23 V versus reversible hydrogen electrode (RHE) in neutral electrolyte solution under AM 1.5 G simulated sunlight (100 mW cm^‐2^), which is 8.4 times higher than that of the CuInSe_2_ photoanode (0.51 mA cm^‐2^). And a benefit of the strong electronic coupling between CISe and N‐doped carbon, the charge transfer rate is increased to 1.3–13 times higher than that of CISe in the range of 0.6–1.1 V versus RHE. The interfacial coupling effects on modulating the carrier transfer dynamics are investigated by Kelvin probe force microscopy analysis and density functional theory calculation. This work provides new insights into bulk phase carrier modulation to improve the performance of photoanode for PEC water splitting.

## Graphical abstract

Inspired by capacitors, well‐defined CuInSe_2_/N‐doped carbon nanorod arrays are designed as the reservoirs to capture and store the photogenerated electrons of CuInSe_2_ by the N‐doped carbon, and the strong interface coupling can pave a specific “bridge” between components to promote carrier migration and separation for PEC water splitting.

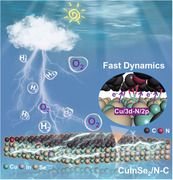

## 1 Introduction

Photoelectrochemical (PEC) water splitting for hydrogen generation is of great significance in the field of carbon‐neutral technology for zero‐emission renewable energy evolution. ^[^ ^1^, ^2^ ^]^ To scale up H_2_ evolution reaction, the photoanode with high bulk charge separation efficiency is in the first level task, which is unfortunately difficult for most semiconductors. The construction of heterojunctions by coupling different materials with diverse energy level structure including type II heterojunction, Z‐type heterojunction, and S‐type heterojunction have been regarded as one of the most promising strategy to improve the electron‐hole separation, and affording a specific redox potential for the catalytic reaction. ^[^ ^3^, ^4^, ^5^ ^]^ However, it is still faces serious challenges originating from the unreasonable energy band structure, and large interface energy resistance, which inhibit the spontaneous migration and separation of photogenerated carriers. ^[^ ^6^ ^]^Accordingly, it is highly desirable to design a suitable interface structure with spontaneous carrier migration for efficient solar water splitting.

Ternary metal selenide or chalcogenide semiconductors, such as ZnIn_2_S_4_, CuInSe_2_ have emerged as one of the most promising candidates for photocatalytic water splitting. CuInSe_2_ (CISe) is a typical n‐type semiconductor with adjustable bandgap of 1.0–2.0 eV. The maximum value of the conduction band position is as high as about ‐1.0 eV, which endows the strong reducing capacity for the photogenerated electrons.^[^ ^7^, ^8^ ^]^ The bulk carrier recombination in CISe seriously limits its photo‐to‐electric conversion efficiency. The strategy of coupling suitable materials with CISe to build the interfacial heterojunction is thought to be a feasible way to regulate the carrier spontaneous migration. Carbon material with *π*‐conjugated structure presents an attractive electron mobility, tunable electronic local structure, and good catalytic economical applicability.^[^ ^9^, ^10^, ^11^ ^]^ Some pioneer composites including activated Carbon‐TiO_2_^[^ ^12^ ^]^ carbon spheres‐BiVO_4_,^[^ ^13^ ^]^ N‐C/In_2_O_3_‐CuO,^[^ ^14^ ^]^ In_2_Se_3_@N‐C ^[^ ^15^ ^]^ have been proposed for improving their photocatalytic performances. As electron acceptor, the carbon material could capture the photoinduced electrons, whereas, the lack of a tight interaction between the carbon material and the semiconductor interface can weaken the ability to capture electrons due to the high energy barriers at the interface. The continuous distribution of carbon in the composite and their interface contact are the current big questions. The poor interfacial contact is like stacking a “wall” between the composites, seriously preventing the trajection of charge flow. The intimate interface combination, such as in strong chemical bonds, could pave a specific “bridge” to promote carrier migration between components, and facilitate modulating the interface electronic structure and space charge density distribution.^[^ ^16^, ^17^ ^]^ We also have reported that engineering the heterogeneous interfaces via in‐situ reaction protocols could largely decrease the phase boundary thermal resistance for the migration and separation of electron and holes.^[^ ^18^, ^19^ ^]^ To this end, constructing carbon continuous distribution in CISe with intimate interfacial contact might be an effective approach to regulate the charge spontaneous migration and separation for highly efficient PEC water splitting.

Electric double‐layer capacitor is an efficient energy storage device for the uneven power demand in sustainable energy systems.^[^ ^20^, ^21^, ^22^ ^]^ It works as follows: the carbon electrode adsorbs electrons in the charging stage, and then release electrons in the discharging stage. Inspired by this, we reported the CuInSe_2_/N‐doped carbon (CISe/N‐C) nanorod arrays by using the N‐doped carbon as the reservoirs to capture and store the photogenerated electrons of CISe. The strong interfacial coupling between CISe and N‐doped carbon could facilitate the migration and separation of electrons and holes. The typical CISe/N‐C photoanode shows a maximum photocurrent density of 4.28 mA cm^‐2^ at 1.23 V versus reversible hydrogen electrode (RHE) in neutral electrolyte solution under AM 1.5 G simulated sunlight (100 mW cm^‐2^), an 8.4 times enhancement relative to that of the CISe sample. In addition, the photo‐stability is also greatly improved in the redox electrolyte solution, 98.11% initial value is maintained even after 3600 s continuous light illumination at an applied potential of 1.0 V versus RHE. As compared to the similar photoelectrodes, the activity of the CISe/N‐C sample is superior to the most reports in recent literature. The prominent performances of the CISe/N‐C photoanode can be ascribed to the high carrier separation efficiency, low interfacial resistance driven by the berried continuous electrons reservoirs, and the strong interfacial coupling interactions, the charge separation mechanism of which is understood from the electrochemical analysis and the density functional theory (DFT) simulation. This work might be important for understanding the interfacial charge migration within nanostructured photoelectrodes boosting the high PEC applications.

## 2 Results and Discussion

**Figure 1:**
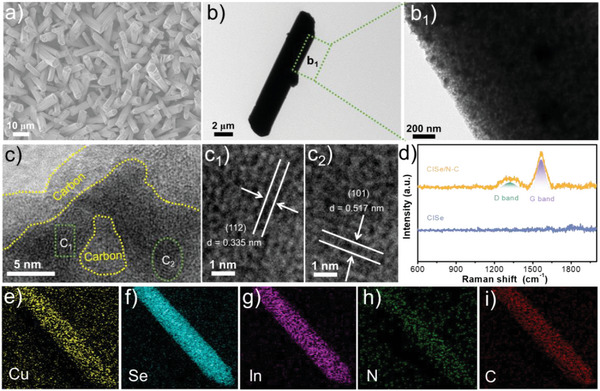
SEM image (a), TEM images (b) and the magnified region (b_1_), HRTEM image (c), and the magnified regions (c_1_, c_2_), Raman spectra (d), and EDX mapping images (e‐i) of Cu (yellow), Se (light green), In (purple), N (green) and C (red) for the typical CISe/N‐C sample.

Well‐defined CISe/N‐C nanorod arrays are synthesized via a selenization process of etching Cu/In‐metal organic framework (MOF). As shown in Figure S1 (Supporting Information), the as‐prepared Cu/In‐MOF exhibits nanorod morphology with a hexagonal cross‐section, length of 10‐15 µm, and diameter of 2–5 µm. Interestingly, the CISe/N‐C inherits the pristine Cu/In‐MOF template by a hexagonal nanorod morphology with 8–12 µm in length and 1‐3 µm in diameter (**Figure** **1**a). The hexagonal feature of CISe/N–C can be clearly seen from the cross‐section at the end of the rods. This phenomenon is similar to other reports about applying MOFs as precursors/templates for the synthesis of nanomaterials.^[^ ^15^ ^]^ From TEM images (Figure 1b), it can be seen that the nanorod exhibits a porous external surface with large surface area, which facilitates for the exposure of catalytic active sites and the penetration of electrolyte ion from outside to inner. Additionally, a uniformly distribution of carbon particles is helpful for the electron reservoir. From HRTEM image (Figure 1c), a clear brightness contrast between the shell and the inner core can be seen. The outer bright shell with a thickness of about 5 nm is thought to be the amorphous porous layer. The magnified HRTEM images (Figure 1c) indicate that the inner dark regions with clear lattice fringes of 0.335 nm and 0.517 nm are well indexed to the (112) and (101) lattice plane of chalcopyrite CuInSe_2_ (JCPDS 40‐1487).^[^ ^23^ ^]^ The Raman spectra (Figure 1d; Figure S1c, Supporting Information) displays a D band at 1325 cm^‐1^ and a G band at 1567 cm^‐1^, which further confirms the presence of carbon component in the products.^[^ ^15^ ^]^ In addition, CISe does not exhibit similar characteristic peaks, implying that carbon formation can be controlled by in situ annealing. Besides, the elemental mappings of the CISe/N‐C nanorod (Figure 1e‐i) exhibit that the uniformly distribution of Cu, Se, In, N, and C elements in the selected region, revealing the intimate phase contact between the carbon and CISe, which enables the continuous separation of photoinduced electrons and holes.

**Figure 2:**
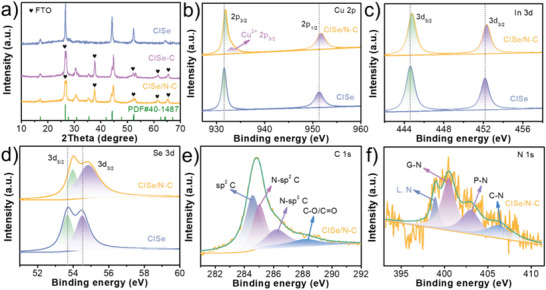
XRD pattern (a), High‐resolution XPS spectra of Cu 2p (b), In 3d (c), Se 3d (d), C 1s (e), and N 1s (f) of the CISe, CISe‐C, and CISe/N‐C samples.

The microstructures of CISe, CISe‐C, and CISe/N‐C samples are characterized by X‐ray diffraction (XRD), X‐ray photoelectron spectroscopy (XPS) analysis. As shown in **Figure** **2**a, all diffraction peaks can be indexed to the chalcopyrite CuInSe_2_ (JCPDS 40‐1487).^[^ ^23^ ^]^ In Cu 2p spectra (Figure 2b), the Cu 2p_3/2_ and 2p_1/2_ peaks are located at 931.6 eV and 951.3 eV for the pristine CISe, respectively. In contrast, Cu 2p_3/2_ and 2p_1/2_ peaks of CISe/N‐C show a slightly positive shift of ca. 0.2 eV and 0.4 eV, respectively. In addition, as shown in Figure 2c,d, compared to the CISe, In (3d_5/2_ 444.8 eV, 3d_3/2_ 452.4 eV) and Se (3d_5/2_ 54.0 eV, 3d_3/2_ 54.9 eV) in CISe/N‐C also exhibit a slight positive shift. It might be aroused by the carbon component, which adsorbs the electron cloud of CISe to its surface, and resulting in the adjacent atoms shift to a high binding energy position.^[^ ^24^ ^]^ The high resolution C 1s spectra (Figure 2e) are composed of four deconvoluted peaks, which are 284.5 eV, 285.1 eV, 286.3 eV, and 288.2 eV in turn, and can be assigned to sp^2^‐C, N‐sp^2^ C, N‐sp^3^ C and C‐O/C=O bonds.^[^ ^25^, ^26^ ^]^ The four peaks of N1s spectra (Figure 2f) can be ascribed to the graphene‐N (400.4 eV), the pyridine‐N atom bond (403.1 eV) and the chemisorbed nitrogen (406.1 eV).^[^ ^15^ ^]^ Notably, the peak at 398.9 eV can be attributed to lattice N, implying a N‐doping in the composites, possibly originating from the pyrolysis of the Cu/In‐MOF precursor.^[^ ^14^, ^27^ ^]^ Meanwhile, the presence of Cu^2+^ (2p_3/2_ 933.1 eV) in CISe/N‐C implies a strong interfacial coupling effect between CISe and N‐doped carbon.

**Figure 3:**
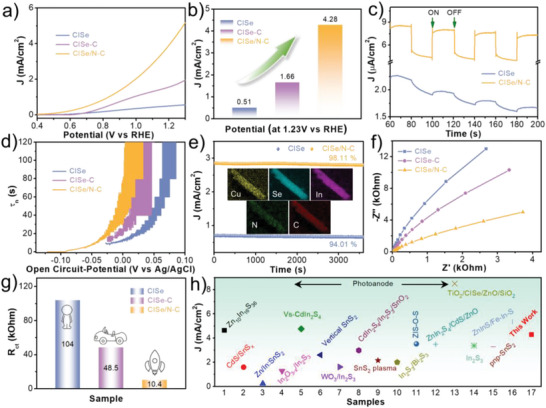
LSV curves and potential at 1.23 V versus RHE current density (a, b), Photocurrent response (c), Electron lifetime (d), Stability tests at 0.25 M Na_2_S and 0.35 M Na_2_SO_3_ (e), EIS results under dark conditions (f), and *R_ct_* (g) of the CISe, CISe‐C, and CISe/N‐C samples, respectively. Detailed comparison of the PEC performances of the CISe/N‐C with the reported literatures (h).

To evaluate the PEC performances, the as‐prepared photoanodes are carried out in 0.5 M Na_2_SO_4_ electrolyte under 1.5G illumination with a standard three‐electrode system. As shown in **Figure** **3**a, the pure CISe sample exhibit extremely low photocurrent density. Although the CISe‐C is an *in‐situ* formed carbon layer, the photocurrent density is only about 1.66 mA cm^‐2^ at 1.23 V versus RHE (Figure 3b) under AM 1.5 G simulated sunlight (100 mW cm^‐2^). However, for the typical CISe/N‐C photoanode, the value gets to as high as 4.28 mA cm^‐2^ at 1.23 V versus RHE and the onset potential also negatively shifted, the value is 8.4 times higher than that of the pure CISe photoanode. Meanwhile, the current density of CISe/N‐C photoanode is also higher than that of CISe in dark (Figure S2a, SI) because of the carbon layer electron trapping effect. The enhancement in photocurrent density might be aroused by the effect of carbon electron capture and the strong interfacial coupling between CISe and N‐doped carbon, which increase the separation of photoinduced electrons and holes, decreases the interface resistance, and promotes the transfer of bulk charges. The annealing times on PEC performances are also investigated as shown in Figure S2b (Supporting Information). As seen, the photocurrent density increased from 0.94 to 4.28 mA cm^‐2^, and dropped to 2.2 mA cm^‐2^ at 1.23 V versus RHE against annealing times. The change might be caused by the interface resistance. Longer times may facilitate the intimate interface contact and promote the carrier transfer. Figure 3c and Figure S2c (Supporting Information) show the photo responses of the as‐prepared photoanodes over time at 0.2 V versus RHE with chopped light illumination. The photocurrent density of the typical CISe/N‐C photoanode is much higher than those contrasting sample, indicating much higher separation efficiency of photoinduced electrons and holes. The carrier lifetime (*τ*_n_) is calculated by the open‐circuit photovoltage measurements in dark as shown in Figure 3d and Figure S2d (Supporting Information). The *τ* value of the CISe/N‐C photoanode is significantly prolonged than those samples, indicating that the interfacial coupling interactions promote the carrier transfer. The stability curves are also used to evaluated the activity of the as‐prepared photoanodes. As seen from Figure 3e and Figure S2e (SI), the photocurrent density of the typical CISe/N‐C photoanode maintains a slightly decay with 98.1% of its initial value under continuous illumination for 1 h. In contrast, the current density retention rate of CISe‐C photoanode is only 87.76%, suggesting seriously photocorrosion occurred by the insufficient interface contact. And the element distribution for the CISe/N‐C photoanode keeps similar characteristics to the initial after high current density (2.85 mA cm^‐2^) test, further demonstrating that the interfacial coupling protocol is a promising way to improve the stability. In addition, CISe/N‐C photoanode still maintains a current retention rate of about 88% even prolonging to 3 h (Figure S2f‐k, SI). To clarify the interfacial coupling on charge transfer, electrochemical impedance spectroscopy (EIS) is performed as exhibited in Figure 3f. Among the samples, the CISe/N‐C photoanode display the smallest arc radius at high frequencies of the Nyquist plots, suggesting the lowest interfacial charge transfer resistance (*R_ct_*). The *R_ct_* values can be calculated from the equivalent circuit model based on the EIS data (Figure 3g). The value is as low as 10.4 kΩ for the CISe/N‐C photoanode in contrast to 104 kΩ of the CISe photoelectrode. Meanwhile, the *R_ct_* of the CISe/N‐C photoanode under light is also the smallest (Figure S3, Supporting Information). The performances of CISe/N‐C photoanode are also compared to the similar photoelectrodes in literature as shown in Figure 3h and Table S1 (Supporting Information). The photocurrent density has a distinct advantage in the neutral electrolyte solution.

**Figure 4:**
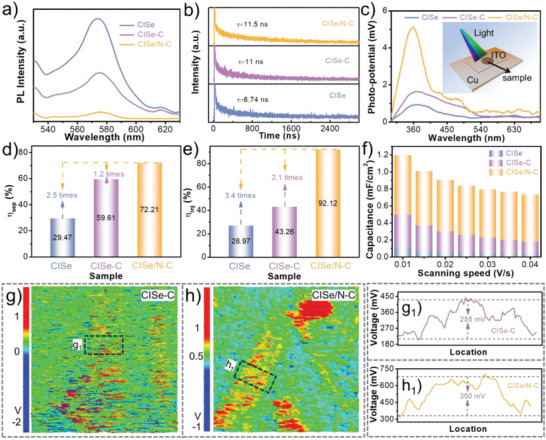
PL spectra (a), time‐resolved PL spectra (b), SPV spectra (c), *η_sep_* and *η_inj_* at 1.23 V versus RHE (d, e), specific capacitance under light irradiation (f) of the CISe, CISe‐C, and CISe/N‐C samples, KPFM image (g, h), and the magnified regions (g_1_, h_1_) of the CISe‐C and CISe/N‐C samples, respectively.

To verify the charge separation, photoluminescence (PL) spectra is performed as shown in **Figure** **4**a. The intensity of the CISe/N–C sample is obviously weaker than those two samples, indicating the excellent ability of N‐doped carbon to trap charges. Time‐resolved PL spectra (Figure 4b) have determined the lifetime of the photogenerated electrons to be 11.5 ns for CISe/N‐C, 11 ns for CISe‐C, and 6.74 ns for pure CISe. A longer lifetime means a higher separation of charge carriers and a more efficient transfer of the photogenerated electrons. Apart from these results, the highest surface photovoltage response intensity of the CISe/N–C sample (Figure 4c) also demonstrates the positive effect of the strong interfacial coupling between N‐doped carbon and CISe on separation of electrons and holes.^[^ ^2^ ^]^ The surface photovoltage of CISe/N–C increases under light illumination for the storage of photogenerated electrons by N‐doped carbon, which is a signal of the “charge” process.

The separation and injection efficiencies of the photoanodes are further calculated from the following equations:

(1) $$\eta_{sep}=\frac{J_{Na_{2}S/Na_{2}SO_{3}}}{J_{abs}}$$

(2) $$\eta_{inj}=\frac{J_{Na_{2}SO_{4}}}{J_{Na_{2}S/Na_{2}SO_{3}}}$$

*J_abs_* obtained from 1.5 G solar spectrum integration:^[^ ^28^ ^]^

(3) $$J_{abs}=\frac{q}{hc}\int_{\lambda }\lambda \phi_{\lambda }\eta_{abs}d\lambda$$

(4) $$\eta_{abs}=\left(1-10^{-A}\right)\times 100\%$$

where the *J_abs_* is the photon adsorption rate, and *q* is the charge of an electron, *h* is the Plank constant, *c* is the light speed, *ϕ_*λ*_* is the photon flux of the AM 1.5 G solar spectrum, and *η_abs_* is the light absorption efficiency. As shown in Figure 4d and Figures S4 and S5 (Supporting Information), the separation efficiency (*η_sep_*) of the CISe is only 29.4% at 1.23 V versus RHE, whereas, the value is as high as 72.21% for the CISe/N–C photoanode. It confirms that the N‐doped carbon as electron reservoir and the strong interfacial coupling effect plays a key role in the migration and separation of charge carriers. The surface injection efficiency of the electrodes also has been evaluated as displayed in Figure 4e. The injection efficiency (*η_inj_*) of the CISe/N–C electrode can reach 92.12% at 1.23 V versus RHE, which is 3.4 times as compared to the pure CISe electrode (26.97%). Cyclic voltammetry (CV) tests are further used to confirm that the N‐doped carbon possesses the ability to extract electrons as displayed in Figure 4f and Figure S6 (SI). As seen, the area of the CV curves for the CISe/N–C electrode gradually increases with the scan rate rising (0.01–0.04 V s^‐1^), and is obviously larger than that of pure CISe electrode in the potential region, indicating that higher capacitance can be obtained in CISe/N–C than in CISe under light irradiation. Furthermore, the capacity of the photoelectrode can be calculated based on the following equation:^[^ ^13^ ^]^

(5) $$C_{s}=\int \left(Id\Psi \right)/\left(2vS\Delta V\right)$$

in which, *I* is the current density of cyclic voltammetry, *ψ* is the potential range, *S* is the working area of photoelectrode, *v* is the scan rate, Δ*V* is the potential window. The photocapacitance of CISe/N‐C is 0.57 mF cm^2^ at 0.03 V s^‐1^, which is 14.6 times higher than that of the CISe (0.039 mF cm^2^). The large capacitance means that the N‐doped carbon can capture more photoinduced electrons from CISe. Under dark conditions, the capacitance of CISe/N‐C is obvious higher than that of CISe, which reflects that the electron spontaneously migrate to the N‐doped carbon layer (Table S2, Supporting Information). Besides, the current density of CISe/N‐C is also higher than that of CISe after electrochemical normalized current comparison (Figure S6h,i Supporting Information). Meanwhile, the Brunauer‐Emmett‐Teller surface area of CISe/N‐C sample (Figure S1d‐f, Supporting Information) is the smallest among the samples, indicating that regulating charge migration via interface engineering is an effective strategy. Kelvin probe force microscopy (KPFM) tests were performed on CISe‐C and CISe/N–C. As shown in Figure 4g,h, CISe/N–C exhibits a higher surface potential, which means that the strong interfacial coupling effect can improve the ability of the N‐doped carbon layer to capture electrons, resulting in more electrons stored in the N‐doped carbon layer. The enlarged KPFM images (Figure 4g,h) show that CISe/N–C has a larger potential difference (350 mV), reflects the enhancement of capacitance caused by strong interfacial coupling.

**Figure 5:**
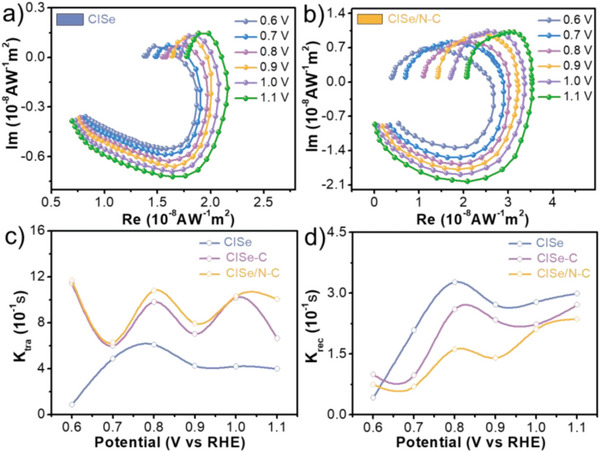
IMPS spectra of the CISe (a) and CISe/N‐C (b) photoanodes, the plot of the rate constant of the *k*_tra,_ (c), and *k*_rec_ (d) versus potential.

The effect of interfacial coupling on charge transfer kinetics for the CISe/N–C electrodes are further discovered by intensity‐modulated photocurrent spectroscopy (IMPS) as shown in **Figure** **5** and Figure S7 (Supporting Information). The negative semicircle of CISe/N–C electrode (Figure 5a,b) is obviously smaller than that of CISe electrode, confirming that a large fraction of the holes arrived to the electrode surface for water oxidation. The transfer rate constant (*k_trans_*) curves of the electrodes at different potentials is shown in Figure 5c, the *k_trans_* value of CISe/N‐C electrode is 1.3‐13 times higher than that of CISe electrode at the potential range of 0.6–1.1 V versus RHE, indicating significantly promoting the bulk charge transport kinetics by the strong interfacial coupling between CISe and N‐doped carbon. The recombination rate constant (*k_rec_*) curves (Figure 5d) gradually upraise with potential increase, suggesting the electron‐hole recombination behavior is largely suppressed even under low bias voltage because of the inner electron storing by the N‐doped carbon within CISe/N‐C electrode.

**Figure 6:**
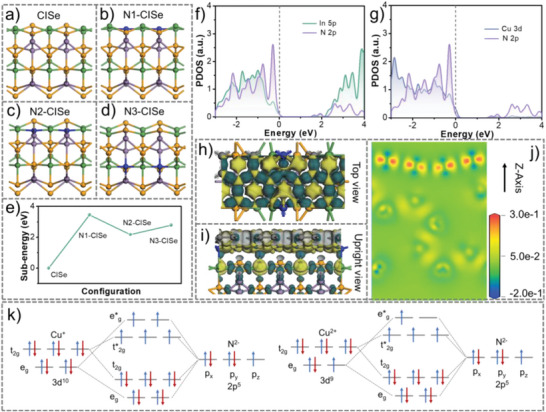
DFT calculations. Structural model for atomic N substituting different atoms in CuInSe_2_: pure CuInSe_2_ (a), surface Se atoms (b), shallow Se atoms (c), deep Se atoms (d), atomic substitution energy (e). PDOS of the N2‐CISe: In‐N interactions (f) and Cu‐N interactions (g). charge distribution top view (h), upright view (i), Z‐axis charge distribution (j) of the CISe/N‐C. The blue and yellow regions imply electron accumulation and depletion, respectively. Schematic diagram of molecular orbitals for 3d electrons in Cu and 2p electrons in N elements coordinated at different valences (k).

The migration and separation of photoinduced charge carriers within CISe/N‐C sample is simulated by density functional theory (DFT) calculation as **Figure** **6** and Figure S8 (Supporting Information). CuInSe_2_ with chalcopyrite structure is shown in Figure 6a, and the tendency and behavior mechanism of N substitution are discussed through theoretical calculations.^[^ ^29^ ^]^ Three situations of substitution sites are predicted: surface Se atoms (N1‐CISe, Figure 6b), shallow Se atoms (N2‐CISe, Figure 6c), deep Se atoms (N3‐CISe, Figure 6d). The substitution tendency is predicted by the corresponding substitution energy. The substitution energy of the corresponding position as displayed in Figure 6e. It is obvious that the N atom replaces the shallow Se atom to form N2‐CISe with the lowest substitution energy (2.18 eV). The introduction of heteroatoms in the main body can affect the local states and coordination numbers of the surrounding atoms. The density of states (PDOS) shows that the main contributions of valence band are main from the Cu 3d and Se 4p orbitals, and the conduction band are from Se 4p and In 5p orbitals (Figure S8b, Supporting Information). Meanwhile, N contribution can reduce the electron‐transfer energy barrier by the localized states near the Fermi level. Further analysis (Figure 6f,g) has indicated that the Cu 3d and N 2p orbitals have two effective cross‐overlaps near the Fermi level, indicating a solid Cu‐N bond in the interface between CISe and N‐doped carbon. The role of carbon layer for trapping electrons is investigated. From the differential charge distribution (Figure 6h,i), it can be seen that the electron density near N‐doped carbon surface within CISe/N‐C sample is higher than other regions, indicating that the electron can be redistributed by the effect of carbon storage.^[^ ^30^ ^]^ The detailed isosurface (Figure 6j) confirms that the electrons are trapped and distributed in the N‐doped carbon layer. In contrast, CISe is in the form of electron‐deficient state, and positive charges are mainly located on the VB of CISe. According to the molecular orbital theory, the 3d orbital electrons of Cu and the 2p electrons of N will form atomic degenerate orbitals. Figure 6k shows two kinds of copper ions and N^2‐^ bonding orbitals, Cu^2+^ has empty orbitals receiving electrons in the anti‐bonding orbitals. This shows two functions: Cu^2+^ and N^2‐^ is easier to bond and form a strong interfacial coupling, the empty orbitals provide more opportunities for electron transfer. Thus, it can be considered that the electrons in the CB of CISe can easily transport to the N‐doped carbon via the interfacial Cu–N bond, which should be the factors responsible for the excellent PEC performance.

**Figure 7:**
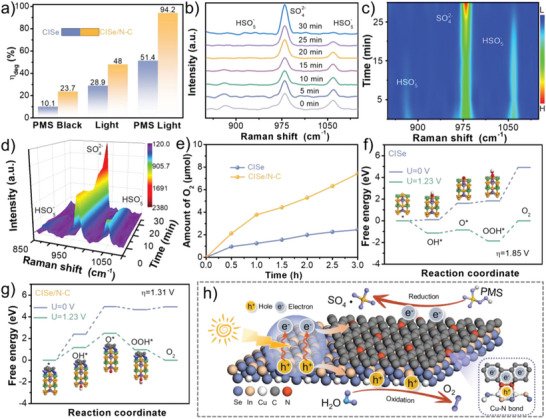
Degradation efficiency under different conditions (a); in situ Raman spectra of the CISe/N‐C (b); 2D plots (top)/contour maps (bottom) (c) and 3D plots (d) of the time‐dependent Raman spectra of the CISe/N‐C; Amount of measured oxygen evolution of the samples (e); Free energies of OER reaction steps for CISe (f), and CISe/N‐C (h); Schematic diagram of the catalytic mechanism (i).

The antibiotic contaminant Adriamycin hydrochloride (DOX) is used as a model pollutant to evaluated the photoactivity of CISe/N‐C sample as shown in **Figure** **7** and Figure S9 (Supporting Information). As reported, peroxymonosulfate (PMS) can be activated by photogenerated electrons to degrade the DOX.^[^ ^31^, ^32^ ^]^ It can be seen that the degradation efficiency of DOX for CISe/N‐C sample is as high as 94.2% within 90 min under visible light illumination (Figure 7a). However, the value for CISe sample is only 51.4%. In situ Raman spectra (Figure 7b), the intensity of HSO_5_^‐^ peak for PMS at 1058 cm^‐1^ gradually weakens, and opposite, the SO_4_^‐^ peak at 980 cm^‐1^ increases. The 2D contour plots (Figure 7c) and the 3D waterfall plots (Figure 7d) further confirm the changes in peak intensity during the activation process. After activation of 30 minutes, an increase in the intensity of SO_4_^‐^ peak can still be detected from the system, verifying that CISe/N–C sample could endow a large number of electrons for activating PMS. The amount of O_2_ generated from the samples was measured using a gas‐tight photoelectrochemical cell (Figure 7e). The O_2_ release of CISe/N‐C is 7.5 µmol, which is three times that of pure CISe, which proves that the electron trapping through N‐doped carbon layer promotes the OER reaction. The energy barrier of the OER reaction is further analyzed by DFT‐free energy.^[^ ^33^, ^34^ ^]^ As shown in Figure 7f, g, the surface overpotential (*η*) value of rate‐determining step (RDS) for the naked CISe from OOH* to O_2_ is as high as 1.85 V, which inhibits the release of oxygen. After the introduction of N‐doped carbon layer, the RDS of CISe/N‐C changes from OH* to O*, indicating that it is favorable for O_2_ production. And the *η* value is obviously reduced to 1.31 V, further indicating that the strong interfacial coupling effect promotes the OER activity of photoanode. The catalytic reaction mechanism is proposed as shown in Figure 7h. Under irradiation, the photoinduced electrons of CISe can be stored by the adjacent N‐doped carbon for the work function difference between CISe and N‐doped carbon. It promoted the separation of bulk charge carriers, like the charge process for a capacitor. However, once the applied bias exceeds the energy barrier, the stored electrons can be quickly transferred to the electrode surface for reducing PMS. The accumulated holes are then consumed through the surface oxygen evolution reaction. It is like the discharge process for a capacitor. By the “charge‐discharge” process, the photoinduced electrons and holes can be efficiently separated and transferred for the PEC reactions.

## 3 Conclusions

In summary, well‐defined CISe/N‐C nanorod arrays have been successfully synthesized via a two‐step method. Like capacitor, the photogenerated electrons of CISe can be stored by the adjacent N‐doped carbon, and the strong interfacial coupling creates direct charge transfer channels between CISe and N‐doped carbon for the charge migration and separation. The optimized CISe/N‐C sample exhibits a maximum photocurrent of 4.28 mA cm^‐2^ at 1.23 V versus RHE in neutral electrolyte solution under AM 1.5 G illumination. DFT calculations suggest that interfacial Cu‐N bonds between CISe and N‐doped carbon efficiently suppress carrier recombination for PEC water splitting. This work provides a rational protocol to modulate the bulk charge migration and separation by interfacial coupling for obtaining highly efficient photoelectrodes.

## 4 Experimental Section

### Synthesis of Cu/In‐MOF nanoarray

Cu/In‐MOF were prepared with some modifications.^[^ ^35^ ^]^ First, 0.193 g Cu(NO_3_)_2_·3H_2_O, 0.481 g In(NO_3_)_3_·xH_2_O, 0.3 g PVP, and 0.185 g PTA were dissolved in a mixed solvent (DMF: EG: H_2_O=2: 2: 1) and stirred for 30 min. Then, 530 µL HNO_3_ was slowly added to the solution. The obtained solution was transferred into a 100 mL Teflon‐lined stainless‐steel autoclave with a piece of clean FTO and maintained at 120 °C for 12 h. After cooling down to the room temperature, the samples were washed with deionized water and ethanol for several times, and dried at 60 °C overnight in air.

### Synthesis of CuInSe_2_/N‐C nanoarray

The obtained Cu/In‐MOF nanoarray and 0.1 g Se powder were placed in the tube furnace with the Se powder in the atmosphere inlet. The temperature was set to 500 °C for 4‐7 h with the increasing rate of 2 °C·min^‐1^ in Ar atmospheres. The synthesized samples were labeled as CISe/N‐C_(x)_, x represents the time. Specially, CISe/N‐C_(6)_ are recorded as CISe/N‐C.

### Synthesis of CuInSe_2_

0.03 g In(NO_3_)_3_·xH_2_O, 0.024 g Cu(NO_3_)_2_·3H_2_O, and 0.0158 g Se powder were dissolved in 20 mL ethylenediamine and 20 mL anhydrous ethanol, then stirring for 60 min. The obtained solution was transferred into a 50 mL Teflon‐lined stainless‐steel autoclave and maintained at 200 °C for 24 h. After cooling down to the room temperature, the samples were washed with deionized water and ethanol for several times, and dried at 60 °C overnight in air. The samples were heat‐treated in argon at 500 °C for 6 h. Specifically, the obtained sample was dispersed in ethanol to form a solution of 10 mg mL^‐1^. Then 0.15 mL was coated on the conductive surface of the glass slide, with a deposition area of about 1 cm × 1 cm. Spin coating speed 800 rpm min^‐1^, spin coating time 30 s, then dry in vacuum at 60 °C. The obtained porous CISe film was exhibited in Figure S10 (SI).

### Synthesis of CuInSe_2_/C

The synthesis process of CISe‐C is similar to that of CISe/N–C, except for the PVP was absent.

### PEC Measurements

The performance of PEC was measured on the electrochemical workstation CHI 660E (CH Instrument Inc., Shanghai) using a three‐electrode system (photoanode, Ag/AgCl as reference electrode, and Pt sheet as counter electrode). The electrolyte was 0.5 m Na_2_SO_4_ aqueous solution (pH 6.8). AM 1.5 G simulated sunlight illumination (100 mW cm^‐2^), which was provided by a solar simulator. Electrochemical impedance spectroscopy (EIS), and intensity‐modulated photocurrent spectroscopy (IMPS) were all measured by Zahner IM6 (Zahner IM6, Germany), in which IMPS was excited by monochromatic light at the excitation wavelength of 455 nm. The stability test was measured at 0.25 m Na_2_S and 0.35 m Na_2_SO_3_ (pH 12.8).

### Oxygen Evolution Detection

Oxygen evolution on photoanodes was measured using a sealed quartz reactor equipped with a typical three‐electrode cell connected to an Ar cycle and a cooling device. In this cell, 0.5 M Na_2_SO_4_ and a 300 W Xe lamp (100 mW cm^‐2^) at an applied bias of 1.2 V versus RHE. The electrolyte was previously purged with Ar for 30 min to remove any dissolved oxygen and cooling system maintained a 4 °C temperature in the reaction device. The PEC rate was determined by measuring the O_2_ created at 30 min intervals.

## Conflict of Interest

The authors declare no conflict of interest.

## Supporting information

Supporting Information

## Data Availability

The data that support the findings of this study are available from the corresponding author upon reasonable request.
