# Supplementary material for: Regulating the Charge Migration in CuInSe2/N‐Doped Carbon Nanorod Arrays via Interfacial Engineering for Boosting Photoelectrochemical Water Splitting
Source: Adv Sci (Weinh). 2023 Apr 23;10(18):2300034. doi: 10.1002/advs.202300034 (PMC10288260; doi:10.1002/advs.202300034)
Supplement: Supplementary file 1 — Supporting Information [file ADVS-10-2300034-s001.pdf]

Supporting Information

Regulating the Charge Migration in $CuInSe_2$/N-doped Carbon Nanorod Arrays via Interfacial Engineering for Boosting Photoelectrochemical Water Splitting

Cheng Wang, Shengdong Sun, Hui Zhang, Jun Zhang, Chuanhao Li*, Wei Chen, Shikuo Li**

Dr. C. Wang, Dr. S. Sun, Prof. H. Zhang, Dr. J. Zhang, Dr. W. Chen, Prof. S. Li

Photoelectric Conversion Energy Materials and Devices Key Laboratory of Anhui Province, Key Laboratory of Structure and Functional Regulation of Hybrid Materials (Anhui University) Ministry of Education, School of Material Science and Engineering & School of Chemical and Chemical Engineering, Anhui University, Hefei, 230601, P. R. China.

Tel.: +86-551-63861328.

E-mail: lishikuo@ahu.edu.cn, zhhui@ahu.edu.cn;

Prof. C. Li

School of Environmental Science and Engineering, Sun Yat-sen University, Guangzhou 510006, China

E-mail: lichuanh3@mail.sysu.edu.cn

1. Experimental section

Chemical reagents: Copper nitrate ($Cu(NO_3)_2·3H_2O$), Polyvinylpyrrolidone (PVP, $(C_6H_9NO)_n$), selenium powder (Se), N,N-dimethylformamide (DMF, $HCON(CH_3)_2$), Nitric acid (HNO_3), and Anhydrous ethanol (C_2H_6O) were purchased from Sinopharm Chemical Reagent Co., Ltd. Indium nitrate hydrate ($In(NO_3)_3·XH_2O$) was purchased from Shanghai Macklin Biochemical Technology Co., Ltd. Terephthalic acid (PTA, $C_8H_6O_4$), Doxorubicin Hydrochloride (DOX, $C_9H_6O_2$), Ethylenediamine ($C_2H_8N_2·H_2O$), Peroxymonosulfate (PMS, $KHSO_5·0.5KHSO_4·0.5K_2SO_4$) were purchased from Shanghai Aladdin Biochemical Technology Co., Ltd. All chemicals were used without further purification. Conductive glass (FTO, 14 Ω, 20 mm×15 mm) was provided by Wuhan Lattice Solar Technology Co., Ltd. High-purity argon (Ar) was purchased from Nanjing Shangyuan Industrial Gas Factory.

Materials characterization: The morphology of the samples was visualized by scanning electron microscopy (SEM, Sigma500, Germany). The lattice structure information of the photoanode was confirmed by transmission electron microscope (TEM) and high-resolution TEM (HRTEM, JEM-2100, Japan). The X-ray diffraction (XRD) pattern of the sample was evaluated by graphite monochromatic Cu K α radiation (λ=1.54056 Å) on an X-ray polycrystal diffractometer (Smart Lab 9KW, Japan). Raman spectra were obtained by laser confocal Raman spectrometer (inVia-Reflex, UK) at room temperature. The chemical state of the surface elements of the sample was measured by X-ray photoelectron spectroscopy (XPS) (ESCALAB 250 Xijing, USA, The XPS data are corrected according to C1s 284.8 eV before analysis). The light absorption of the sample was evaluated by ultraviolet-visible spectrophotometer (UV-3600, Japan). Room temperature photoluminescence (PL) spectra were obtained by FS5 fluorescence (Edinburgh Instruments, UK). Time-resolved transient photoluminescence (TRPL) spectra were measured at Horiba Fluoro max plus (Horiba FluoroMax+, USA). Kelvin probe force microscope (KPFM) measurement on Park atomic force microscope (ParkNX10, Korea). The specific surface area was obtained by a fully automatic MultiTaction specific surface area

analyzer (ASAP-2460, USA).

Open-circuit photopotential (OCP) transient decay and cyclic voltammogram (CV) were performed under the same experimental conditions as the PEC measurements. The OCP decay life can be calculated by the following formula:[1]

$$\tau_n = -\frac{k_B T}{q}\left(\frac{dV_{oc}}{dt}\right)^{-1} \tag{S1}$$

where τ_n is the carrier lifetime, k_B is Boltzmann's constant, T is the temperature, and q is the positive charge.

Electron sacrificial agents were used containing 5 mM $K_3[Fe(CN)_6]$/0.5 M Na_2SO_4, and hole sacrificial agents were 0.25 M Na_2S and 0.35 M Na_2SO_3. Both measured under PEC test system conditions.

The electrochemical surface area-normalized PEC performance was obtained from the following formula:

$$RF = \frac{ECSA}{A_g} \tag{S2}$$

$$j_s = \frac{j_g}{RF} \tag{S3}$$

in which, RF: roughness coefficient, $ECSA$: electrochemical active area, A_g: the electrode area, j_s: the electrochemical surface area normalized current, j_g: the geometric area current.

DOX degradation experiment: The DOX concentration standard curve was obtained by fitting the UV absorption curves of 0.1 mM, 0.3 mM, 0.5 mM, 0.7 mM and 0.9 mM. Degradation is to use 15 mL containing 0.15 mM PMS/0.9 mM DOX, maintain the system temperature at 25 °C. Equipped with an irradiation light intensity of 100 mW cm^{-3} and a piece of FTO (containing the sample). Finally, the degradation efficiency was determined by the UV absorption intensity.

In situ Raman testing: In situ Raman spectroscopy was carried out on a 532 nm laser confocal Raman spectrometer. Pt, Ag/AgCl and copper plate electrodes coated with catalysts are used as counter electrodes, reference electrodes and working electrodes, respectively. Under the applied potential of OCP, the Raman spectra were recorded at

different electrolysis time of 0-30 min. Among them, the illumination is stopped during the Raman test, otherwise the irradiated light intensity of 100 mW cm^{-3} is applied.

Calculation details: All calculations are carried out using CASTEP under the spin polarized density functional theory (DFT) scheme. [2] Specifically, the exchange function is treated by the generalized gradient approximation (GGA) of the Perdew-Burke-Ernzerhof (PBE) function. [2,3] The valence wave function is expanded through the plane wave, the cutoff energy is 500 eV, the geometric convergence tolerance is set to the most powerful 0.03 eV/Å, the maximum energy change 10^{-5} eV/atom, the maximum displacement 0.001 Å and the maximum stress 0.5 GPa. In order to avoid the interaction between periodic images, the distance of vacuum space is set to 15 Å.

The key reaction steps in OER: based on previous studies on the OER pathway and the relevant reaction energies were as follows:

$$H_2O + * \rightarrow OH* + H^+ + e^- \qquad \text{(S4)}$$

$$OH* \rightarrow O* + H^+ + e^- \qquad \text{(S5)}$$

$$O* + H_2O \rightarrow OOH* + H^+ + e^- \qquad \text{(S6)}$$

$$OOH* \rightarrow * + O_2 \uparrow + H^+ + e^- \qquad \text{(S7)}$$

The “*” indicates the active site. “*OH”, “*O”, and “*OOH” represent the intermediate species adsorbed on the active sites. To evaluate the OER activity, the Gibbs free energies (ΔG) are calculated as follows:

$$G = E + E_{ZPE} - TS - \mathrm{e}U \qquad \text{(S8)}$$

where E, E_{ZPE}, and S represent the single-point energy, zero-point energy, and entropy of the CISe and CISe/N-C, respectively, with and without different oxygen intermediates adsorbed. U is the potential compared to a typical hydrogen electrode. The value of T was 298.15 K.

The overpotential (η) for the OER process are calculated as follows:

$$\eta = \frac{\max\{\Delta G_1, \Delta G_2, \Delta G_3, \Delta G_4\}}{e} - 1.23 \quad \text{(S9)}$$

Here, ΔG1, ΔG2, ΔG3, and ΔG4 denote the Gibbs free energy difference for each reaction, (S4)-(S7), respectively.

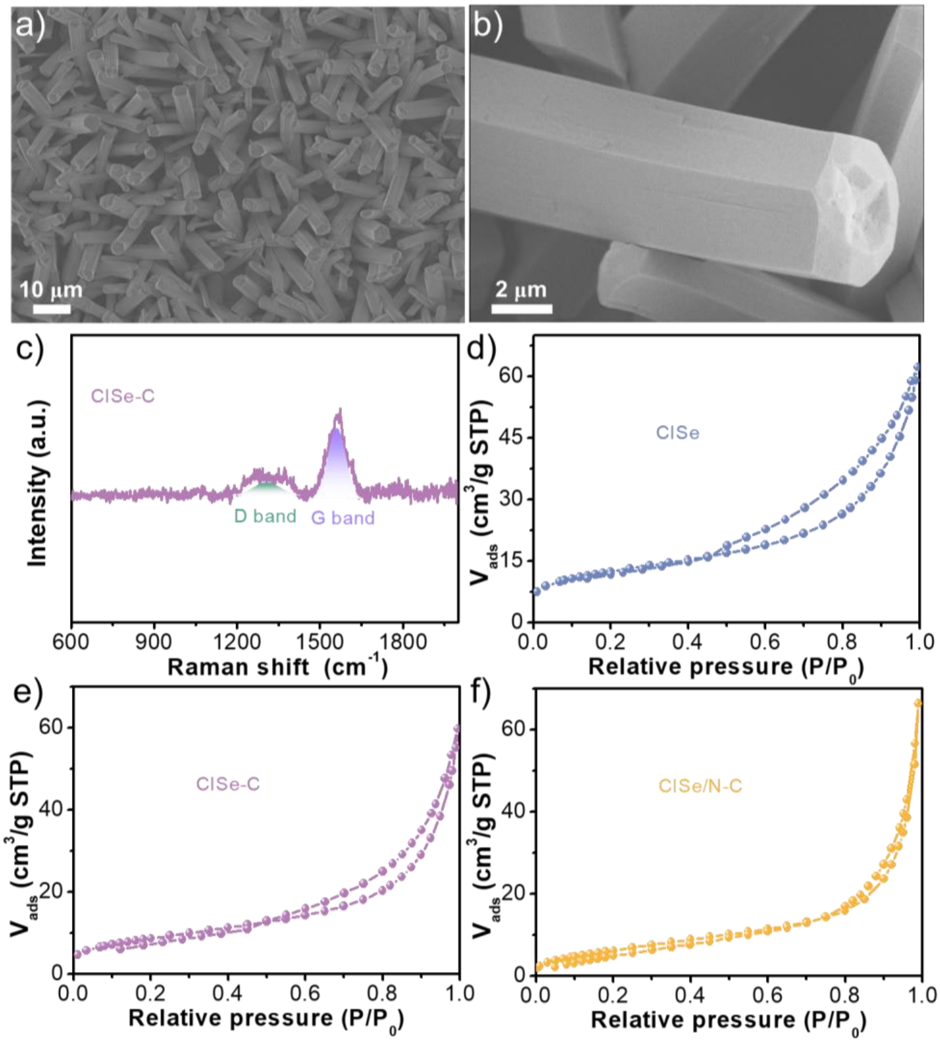

Figure S1. SEM images of Cu/In-MOF (a, b) ; Raman spectra of CISe-C (c); N_2 adsorption-desorption curves of CISe (d) , CISe-C (e), and the CISe/N-C (f) with BET surface area of 43.0 m^2 g^{-1}, 31.2 m^2 g^{-1}, and 24.9 m^2 g^{-1}, respectively.

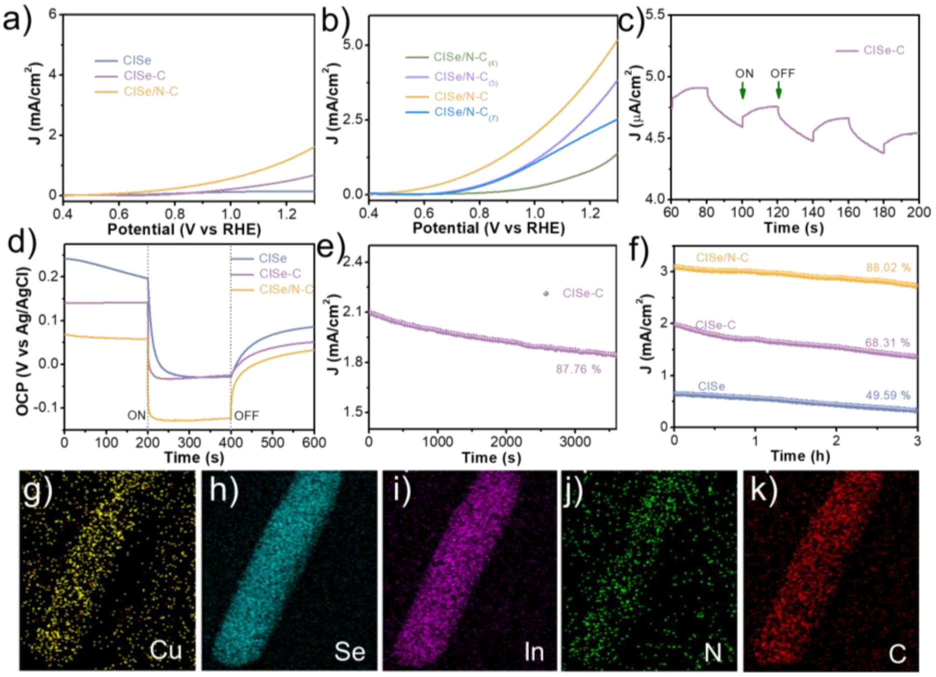

Figure S2. LSV curves of the different photoanodes under dark (a), and different annealing time under light irradiation (b), photocurrent response (c), OCP curves (d), stability tests for 1 hour (e) and for 3 hours (f) of the samples; and EDX mapping images (g-k) of Cu (yellow), Se (light green), In (purple), N (green) and C (red) for the typical CISe/N-C sample after stability tests for 3 hours.

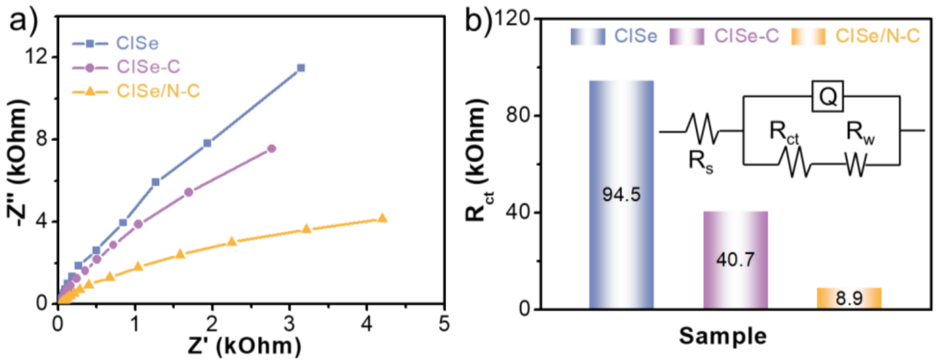

Figure S3. EIS results (a), and R_{ct} (b) of the CISe, CISe-C, and the CISe/N-C samples (light conditions: 100 mW cm^{-2}), respectively.

Note：R_s is the solution impedance, which is carried out in the electrolyte solution. R_{ct} is the charge transfer impedance, which mainly characterizes the characteristics of charge transfer inside the photoelectrode. R_w is the Warburg impedance (diffusion impedance). The electrode reaction is affected by the charge transfer and diffusion, and the diffusion process mainly occurs on the electrode surface. The R_w is usually used to response the diffusion process as reported.

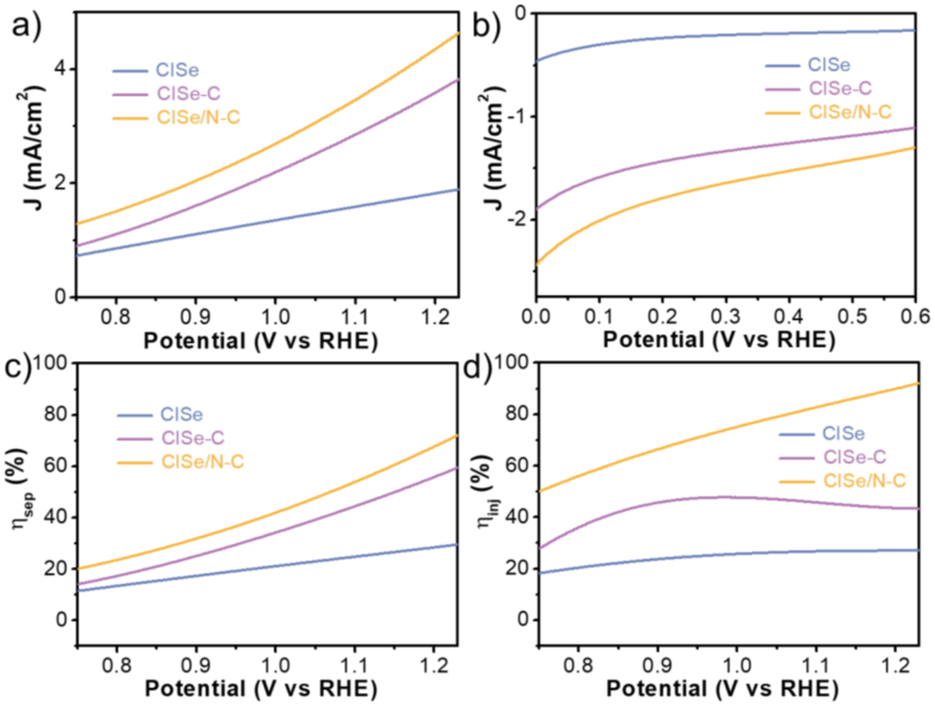

Figure S4. LSV curves of hole scavengers (a) and electron scavengers (b) of the samples; η_{sep} (c) and η_{inj} (d) of the samples.

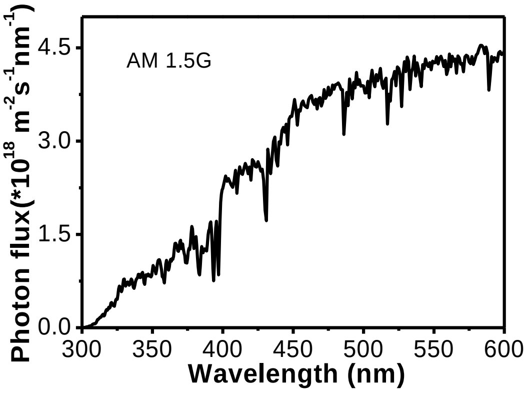

Figure S5. The AM 1.5 G solar spectrum.

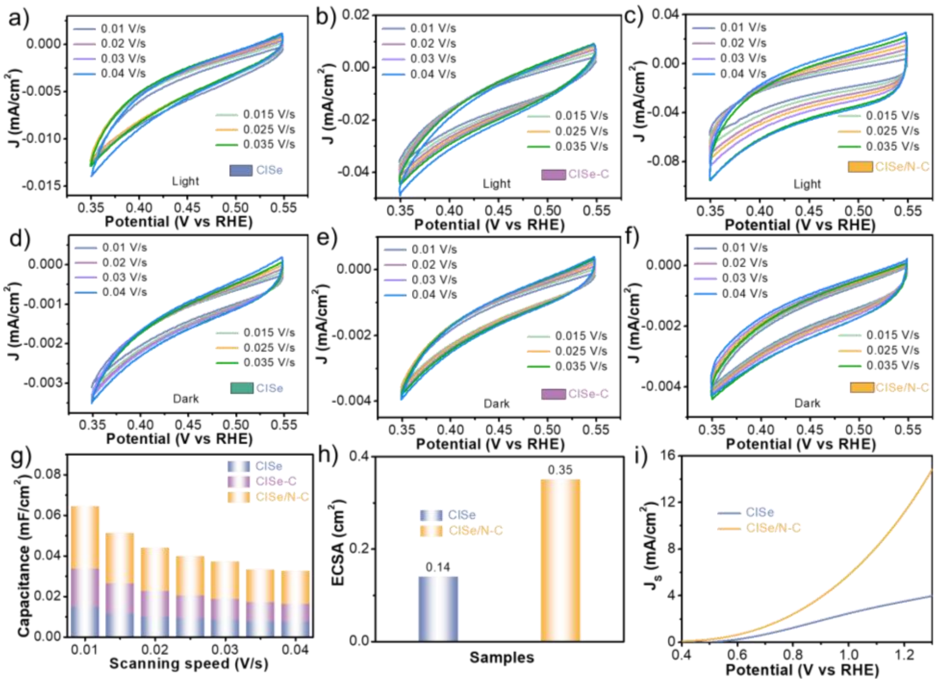

Figure S6. CV curves of CISe (a, d), CISe-C (b, e), CISe/N-C (c, f); specific capacitance under dark (g); ECSA (h) and electrochemical surface area normalized current of the samples (i), respectively (Light conditions: 100 mW cm^{-2}).

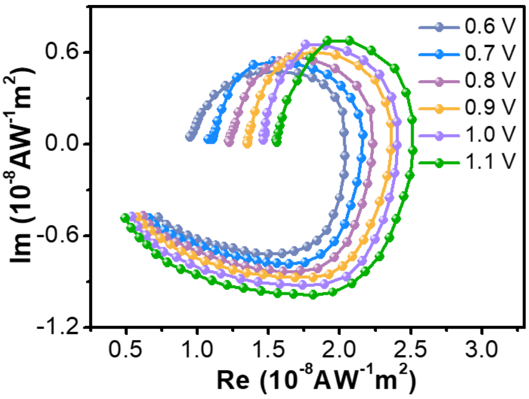

Figure S7. IMPS spectra of the CISe-C photoanode.

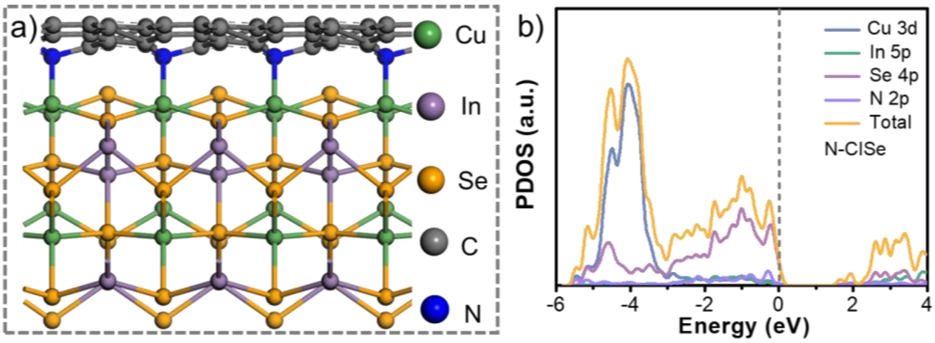

Figure S8. Atomically fabricated structures of the CISe/N-C (a), PDOS of the N2-CISe (b).

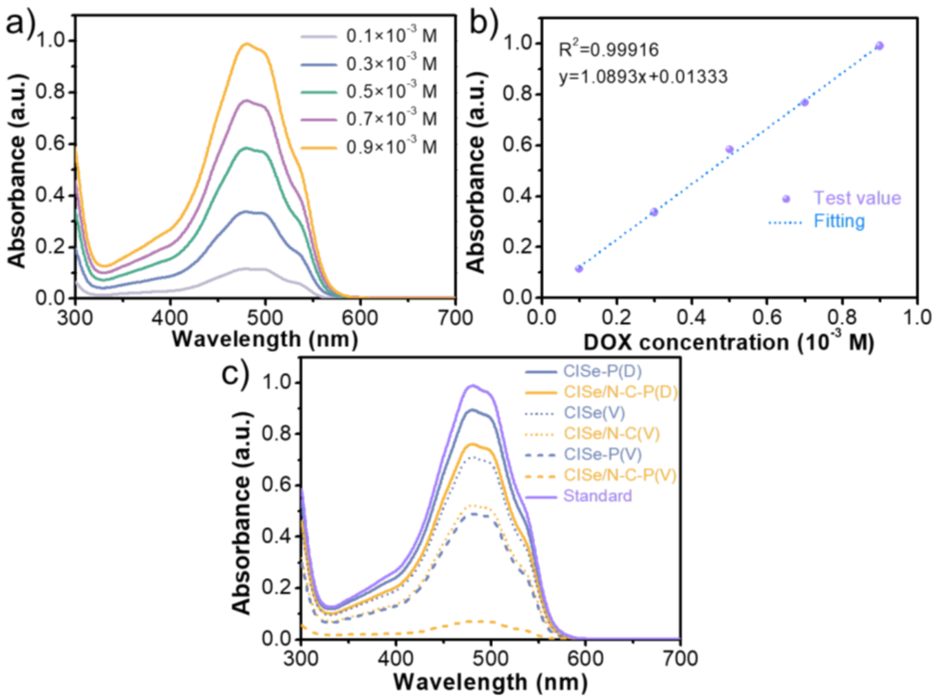

Figure S9. The DOX UV absorption curves of different concentrations (a) and fitting curves (b); degraded UV absorption curve (c).

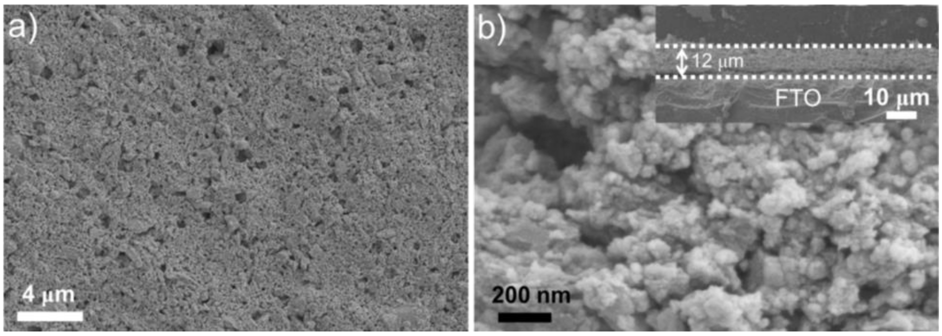

Figure S10. SEM images of the pure CISe film prepared by spin coating.

Table S1. The J of the N-C/CISe in comparison with other photoanodes.

Entry	Photoanodes	J (mA cm^{-2}) at 1.23 V_{RHE}	Electrolyte	References
1	$Zn_{10}In_{16}S_{36}$	4.63	Na_2SO_3	***Angew. Chem. Int. Ed.*** 2018, 57,16882
2	CdS/SnS_x	1.59	Na_2SO_4	***Adv. Funct. Mater.*** 2018, 28, 1706785.
3	Zn/In:SnS_2	0.23	Na_2SO_4	***Angew. Chem. Int. Ed.*** 2019,58, 6761.
4	In_2O_{3-x}/In_2S_3	1.28	KOH	***Adv. Energy Mater.*** 2018,8, 1701114.
5	Vs-$CdIn_2S_4$	5.73	Na_2SO_3	***Nat. Commun.*** 2020,11, 3078.
6	Vertical SnS_2	2.6	Na_2SO_4	***Adv. Energy Mater.*** 2019, 9, 1901236.
7	WO_3/In_2S_3	1.61	Na_2SO_4	***Adv. Energy Mater.*** 2020, 10,1903951.
8	$CdIn_2S_4$/In_2S_3/SnO_2	2.98	Na_2SO_4	***Adv. Mater.****2020*, 32, 2002893.
9	SnS_2 plasma	2.15	Na_2SO_4	***Angew. Chem. Int. Ed.*** 2019, 58, 16668.
10	In_2S_3/Bi_2S_3	2.0	Na_2SO_4	***J. Mater. Chem****. A* 2020, 8, 5612.
11	ZIS-O-S	3.52	NaH_2PO_4/Na_2HPO_4	***Adv. Energy Mater.****,* 2021: 2101181.
12	$ZnIn_2S_4$/CdS/ZnO	3.48	Na_2SO_4	***Adv. Energy Mater.,*** 2021, 11(8): 2003500.
13	TiO_2/CISe/ZnS/SiO_2	ca. 8.5 (at 0.5 V_{RHE})	Na_2S/Na_2SO_3	***ACS Appl. Mater. Interfaces,*** 2022, 14, 603-610
14	In_2S_3	3.37	Na_2S/Na_2SO_3	***Adv. Energy Mater.*** 2020, 10,1902935.

15	pnp-SnS_2	3.28	Na_2SO_4	***Angew. Chem. Int. Ed.*** 2021, 60, 3487.
16	ZnInS/Fe-In-S	5.35	Na_2SO_4	***Nat. Commun.*** 2021, 12, 5247.
17	CISe/N-C	4.28	Na_2SO_4	This Work

Table S2. Capacitance statistics under different conditions.

**Sample** (mF cm^{-2})	**0.01 V/s**	**0.015 V/s**	**0.02 V/s**	**0.025 V/s**	**0.03 V/s**	**0.035 V/s**	**0.04 V/s**	**Condition**
CISe	0.1028	0.0694	0.0537	0.0446	0.0390	0.0350	0.0339	Light
CISe-C	0.3927	0.3011	0.2475	0.2133	0.1879	0.1664	0.1464	
CISe/N-C	0.6997	0.6387	0.6062	0.5819	0.5675	0.5651	0.5543	
CISe	0.0148	0.0116	0.0100	0.0094	0.0086	0.0077	0.0075	Dark
CISe-C	0.0189	0.0149	0.0127	0.0112	0.0102	0.0094	0.0089	
CISe/N-C	0.0308	0.0247	0.0212	0.0193	0.0184	0.0162	0.0162	

References

[1] X. W. Zhang, P. Wang, X. Y. Lv, X. Y. Niu, X. Y. Lin, S. X. Zhong, D. M. Wang, H. J. Lin, J. R. Chen, S. Bai, *ACS Catal*. **2022**, *12*, 2569.

[2] G. C. Zuo, Y. T. Wang, W. L. Teo, A. M. Xie, Y. Guo, Y. X. Dai, W. Q. Zhou, D. Jana, Q. M. Xian, W. Dong, Y. L. Zhao, *Angew. Chem.-Int. Edit*. **2020**, *59*, 11287.

[3] Y. G. Chao, P. Zhou, J. P. Lai, W. Y. Zhang, H. W. Yang, S. Y. Lu, H. Chen, K. Yin, M. G. Li, L. Tao, C. S. Shang, M. P. Tong, S. J. Guo, *Adv. Funct. Mater.* **2021**, *31*, 2100923.
